# The role of PS 18:0/18:1 in membrane function

**DOI:** 10.1038/s41467-019-10711-1

**Published:** 2019-06-21

**Authors:** Tore Skotland, Kirsten Sandvig

**Affiliations:** 1 Department of Molecular Cell Biology, Institute for Cancer Research, Oslo University Hospital, Ullernchausséen 70, 0379 Oslo, Norway; 2 Department of Biosciences, University of Oslo, 0316 Oslo, Norway

**Keywords:** Lipids, Membrane trafficking

## Abstract

Various studies have demonstrated that the two leaflets of cellular membranes interact, potentially through so-called interdigitation between the fatty acyl groups. While the molecular mechanism underlying interleaflet coupling remains to be fully understood, recent results suggest interactions between the very-long-chain sphingolipids in the outer leaflet, and phosphatidylserine PS18:0/18:1 in the inner leaflet, and an important role for cholesterol for these interactions. Here we review the evidence that cross-linking of sphingolipids may result in clustering of phosphatidylserine and transfer of signals to the cytosol. Although much remains to be uncovered, the molecular properties and abundance of PS 18:0/18:1 suggest a unique role for this lipid.

## Abstract (web summary)

There are several lines of evidence for interactions between the two membrane leaflets in cells. In this review the authors discuss the transmembrane coupling of lipids, the involvement of phosphatidyl serine species PS 18:0/18:1, and their importance for various cellular processes.

## Introduction

**Fig. 1:**
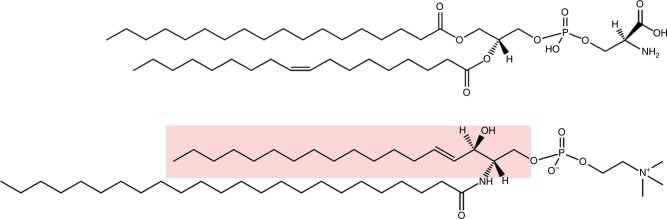
Structures of glycerophospholipids and sphingolipids. The lipids shown are PS 18:0/18:1 (top) and SM d18:1/24:0 (bottom). Different head groups are giving rise to the various lipid classes (Table 1) and different fatty acyl groups result in various species within the class. Note that sphingolipids often contain N-amidated fatty acyl groups much longer than those most common in glycerophospholipids. The sphingoid base of SM d18:1/24:0 is highlighted in red. It should be noted that the double bond C18:1 (oleic acid) is in cis configuration and that the double bonds of all fatty acyl groups of phospholipids and sphingolipids have a cis configuration. The structures have been made by using the structure drawing tools available at Lipid Maps (www.lipidmaps.org)

Lipids are grouped into different classes mainly based on their head groups, whereas various hydrophobic chains give rise to different species within the class (Table 1 and Fig. 1). It is estimated that cells comprise several thousands lipid species, whereas current mass spectrometry (MS) based analyses allow us to quantify ~1000 species^1–3^. Glycerophospholipids are the main constituents of cell membranes. Their amphiphilic character is due to the hydrophilic head and the hydrophobic fatty acyl tails. Generally referred to as phospholipids, glycerophospholipids can form a bilayer. Cellular membranes are such bilayers, where the hydrophobic tails of the phospholipids are located in the middle of the membrane with the hydrophilic head groups facing the hydrophilic surroundings on both sides. Most phospholipids contain ester-bound fatty acyl groups (although also ether-linked phospholipids are common in most tissues) with 16 or 18 carbon atoms and zero, one or two double bonds in cis configuration. However, other fatty acyl groups like C20:4 (arachidonic acid) and C22:6 (docosahexanoic acid; DHA) are also common in the *sn-2* position. In this review much of the emphasis will be put on phosphatidylserine (PS), which is a phospholipid with serine bound via a phosphodiesther linkage to glycerol (Fig. 1). For a new and detailed review article about the diversity of membrane lipid composition, see ref. ^4^.

**Table 1:** The most common lipid classes in membranes

Lipid class/abbreviation	R1	R2	Headgroup
Phosphatidylcholine/PC	FA	FA	Choline
Lysophosphatidylcholine/LPC^a^	FA	H	
Ether-linked PC: PC O, PC P^a^	Alkyl, alkenyl	FA	
Phosphatidylserine/PS	FA	FA	Serine
Phosphatidylethanolamine/PE	FA	FA	Ethanolamine
Phosphatidylinositol/PI	FA	FA	Inositol
Phosphatidylglycerol/PG	FA	FA	Glycerol
Phosphatidic acid/PA	FA	FA	H
Sphingomyelin/SM	LCB	FA	Phosphocholine
Glycosphingolipids/GSL^b^	LCB	FA	Carbohydrates

*FA*   fatty acyl group, *LCB* long-chain base

^a^Lysolipids and ether-linked lipids may occur in several lipid classes, but are for simplicity shown for PC only

^b^Structures of the high number of glycosphingolipid classes (including Gb3 and GM1 discussed here) are shown in ref. ^5^.

**Fig. 2:**
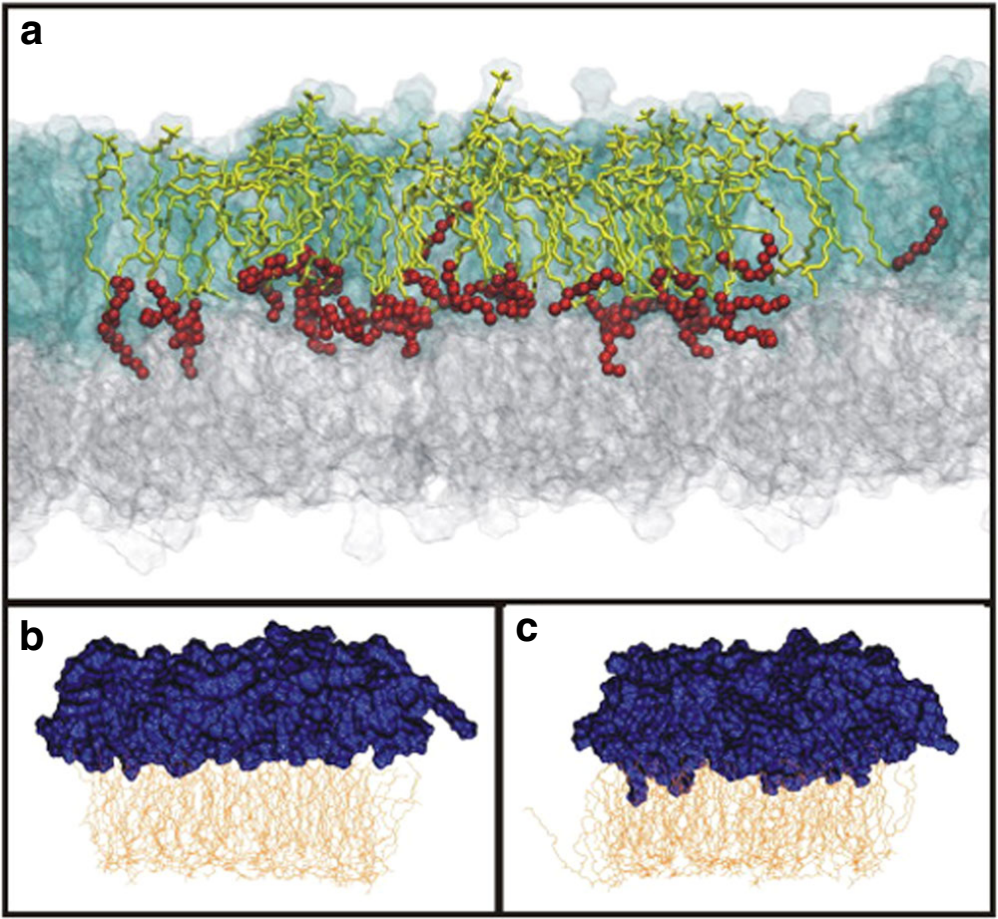
Illustration of interdigitation between the two membrane leaflets. **a** Multicomponent bilayer where SM d18:1/24:0 molecules are shown as yellow sticks with the last eight carbon atoms depicted as red balls. Lipids in the outer leaflet are shown as transparent blue glass, while lipids in the inner leaflet are described as transparent grey glass. For clarity SM d18:1/24:0 molecules are marked only in the central part. **b** Model for a bilayer with PS 18:0/18:1 and cholesterol in the inner leaflet and SM d18:1/16:0 and cholesterol in the outer leaflet. **c** Similar to B, but SM d18:1/16:0 has been exchanged with SM d18:1/24:0. Blue colour is used for the outer leaflet and yellow for the inner monolayer. Note that the N-amidated fatty acyl group med 24 carbon atoms (in C) are penetrating deeper into the opposite leaflet than the species with 16 carbon atoms (in **b**). For more details see ref. ^52^, from where this figure is reproduced

Sphingolipids are also present in cellular membranes but are different from the phospholipids in that their backbone is composed of a sphingoid base (Fig. 1). Whereas the phospholipids are synthesised from glycerol, the sphingolipids are synthesised from serine. The sphingolipids contain N-amidated fatty acyl groups (N originating from serine) that often are much longer than the fatty acyl groups of the phospholipids (Fig. 1). The sphingolipids frequently have a “bimodal” species distribution, meaning that they contain mainly N-amidated C16:0 as the shortest species and C22-C24 as the longest species^5–9^, with very little present of N-amidated C18 and C20 species. In cells the major sphingolipids species are C16:0, C24:0 or C24:1^10,11^ (more details are given below). The reason for this bimodal distribution is not known. For the purpose of the present discussion it should be noted that C16 and C18 hydrophobic chains are able to reach approximately to the middle of the membrane bilayer, whereas C24 chains are so long that they should be able to reach far into the opposite layer (Fig. 2) and thus be able to give a larger interleaflet coupling as described below.

### The plasma membrane and the concept of interleaflet coupling

The plasma membrane is known to have an asymmetric distribution of lipids between the two leaflets. Almost all sphingolipids and phosphatidylcholine (PC), a phospholipid with a choline headgroup, are present in the outer leaflet, whereas most of the other plasma membrane phospholipids, such as phosphatidylserine (PS), phosphatidylethanolamine (PE) and phosphatidylinositol (PI), are present in the cytosolic leaflet. It should be noted that cholesterol constitutes 30–40% of all lipids in the plasma membrane. The distribution of cholesterol between the two leaflets have been heavily discussed for many years, but recent data indicate that it is close to evenly distributed in the two leaflets. Although a lot of information about the intracellular distribution of lipids is available^1^, there is still much to learn about both the distribution of the different lipid classes and their species. As discussed in this review there are several lines of evidence for interactions between the two membrane leaflets in cells and the importance of the length of fatty acyl chains (Fig. 2). Here we discuss the available data relating to transmembrane coupling of lipids (the interaction between fatty acyl chains originating from the two opposing leaflets) and its importance for different cellular processes. We especially focus on the evidence suggesting that the phosphatidylserine species PS 18:0/18:1 play a singular role for membrane function.

### Signalling through ligand binding to the outer leaflet

Membrane domains with a high lipid order, including lipid rafts (often referred to as membrane rafts) have been reported to show interactions between the two membrane leaflets and to be important for transmembrane signalling, endocytosis and intracellular transport^12–17^. Such domains are enriched in cholesterol and glycosphingolipids which as mentioned above often have long fatty acyl chains, thus facilitating interactions with the inner leaflet. The binding of multivalent bacterial toxins to glycosphingolipid receptors on host cells results in intracellular signalling^18–23^. This raises the question of how binding of a protein toxin to lipids in the extracellular leaflet of the plasma membrane could result in signalling on the other side of the membrane. One possibility is that toxin-induced signalling is due to cross-linking and clustering of glycolipids.

The bacterially produced Shiga toxin (Stx) and cholera toxin, so called AB5 toxins, contain one enzymatically active A-chain and five B-chains binding to glycosphingolipids on the host cell’s outer membrane. Stx has three binding sites for globotriaosylceramides (Gb3) on each B-chain, but it is not known how many of these binding sites that normally are occupied when Shiga toxin is bound to cells. In contrast, the five B-chains of cholera toxin each bind one monosialotetrahexosylganglioside (GM1); for reviews see^24–27^. Also, the SV40 virus multivalently binds to GM1^28^, and there are additionally several toxins and viruses that require glycosphingolipids to interact with cells and induce signalling^29^.

Consistent with the idea that toxin-induced signalling is due to cross-linking of glycosphingolipids on the plasma membrane, cross-linking of these lipids with antibodies or lectins results in a similar signalling^30–32^. Several possibilities exist for how this might occur. (i) An unknown transmembrane protein could be interacting with the toxins and be involved in this signalling, somewhat similar to what is known for many protein receptors in the plasma membrane. However, no such protein has yet been identified for Stx binding to Gb3 at the cell surface. (ii) The sphingolipids often contain a high fraction of very-long-chain N-amidated fatty acyl groups, e.g. with 22–24 carbon atoms, such that they theoretically should be able to penetrate almost halfway into the inner leaflet. The toxin-induced binding and clustering of these very-long-chain sphingolipids in the outer leaflet by interleaflet coupling could therefore cluster lipids such as PS or phosphorylated phosphatidylinositols (PIPs)^11,33,34^ in the inner leaflet. The accumulation of PS or PIPs would in turn provide an increased avidity for binding of cytosolic proteins and thus possibly initiate a signalling cascade. (iii) The multivalent binding of these toxins to the cell by cross-linking of lipids could change the conformation of a transmembrane protein, and create Ca-channels resulting in intracellular signalling and downstream effects. In such a scenario, transbilayer coupling may be required.

Cholera toxin might trigger intracellular signalling via multiple pathways, since it can also bind to surface glycoproteins^35–37^. Furthermore, GM1 is able to interact with membrane proteins^38^. Thus, we will focus on Shiga toxin binding to its lipid receptor Gb3 to provide a basis for discussing interleaflet coupling.

### How Stx triggers intracellular signalling

Gb3, the lipid receptor for Stx, consists of a mixture of species with different N-amidated fatty acyl groups containing mainly between 16 and 26 carbon atoms^8^. The length of the N-amidated fatty acyl groups of Gb3 has been suggested to play a role not only in the binding to Gb3^25^, but also for transport of Stx to the Golgi apparatus and for the Stx toxicity on cells^39–42^. Thus, there may be a communication between Gb3 in the outer leaflet and the sorting machinery at the cytosolic side of the plasma membrane, e.g., due to interleaflet coupling between Gb3 and lipids in the inner leaflet.

Theoretically, a high fraction of the Gb3 species should be able to reach approximately halfway into the inner leaflet of the plasma membrane and thus contribute to interleaflet coupling^8^. It should be noted that the dominant Gb3 species (C24:1) in HEp-2 cells is the only major Gb3 species with a double bond^10,43^. Since C24:1 (nervonic acid; cis-15) has its double bond between carbon atoms 15 and 16, the double bond of Gb3 d18:1/24:1 will be almost exactly at the centre of the membrane bilayer. Why do biological membranes contain such high amounts of very-long-chain sphingolipids, including one with a double bond placed close to the middle of the bilayer? We speculate that the bimodal distribution of sphingolipids species (mainly C16:0 and the much longer C22:0, C24:0 and C24:1) in cells is important to have such lipids reaching both to the centre of the double layer and far into the other leaflet, thereby creating flexibility for interactions of different strengths between sphingolipids in one leaflet with lipids in the other leaflet.

Gb3 is found both in detergent resistant membranes (DRMs) and non-DRM membrane fractions of many cell lines^44^. Although DRMs as a tool to study lipid rafts is under debate^45^, the lipid composition of DRMs may to some extent reflect the composition in lipid rafts. Different studies, including studies using DRMs, indicate that Gb3 present in high lipid-order membrane fractions such as lipid rafts are involved in retrograde transport (i.e., transport to the Golgi apparatus and ER) and toxicity of Stx following transport of the A-chain to cytosol^25,41^. In particular, nanodomains containing cholesterol were reported to be important for the Stx induced intracellular signalling^18^ since addition of filipin (which binds to cholesterol) had an inhibitory effect, and extraction of cholesterol with methyl-β-cyclodextrin reduced Golgi transport of Stx^46^. One can, however, not exclude that such treatments have unspecific effects.

### Interleaflet coupling between sphingolipids and PS

While many proteins span the membrane bilayer and are important for its stability, we will focus on interleaflet coupling due to the less well understood interactions between lipids in the two leaflets^47,48^. Those interactions are often investigated using model membranes lacking the complexity and asymmetry of biological membranes. To our knowledge the first study where quantification of many lipid species of biological membranes was followed up with molecular simulation, was the investigation of exosomes released from PC-3 cells^11^. Exosomes are small extracellular vesicles released from cells upon fusion of multivesicular bodies (late endosomes) with the plasma membrane. Mass spectrometry (MS) analyses of PC-3 cells and exosomes released from these cells, revealed that cholesterol—the very-long-chain SM species (mainly SM d18:1/24:0 and SM d18:1/24:1) and PS 18:0/18:1 (which is the main PS species making up ~40% of PS species in these exosomes)—were enriched to a very similar extent between cells and exosomes. Furthermore, if one compensates for the different surface areas of the inner and outer leaflet of exosomes with a diameter of 70 nm, PS constitutes approximately half of the phospholipids of the inner leaflet, and PS 18:0/18:1 in the inner leaflet could theoretically cover ~80% of the area covered by the very-long-chain sphingolipids (mainly SM) in the outer leaflet^11^. These data lead to the idea that these lipids might be sorted together due to interactions (“hand-shaking”) between the very-long-chain SM in the outer leaflet and the PS 18:0/18:1 in the inner leaflet^11^.

A follow up study performed with PC-3 cells treated with the ether lipid precursor hexadecylglycerol resulted in major changes in the cell lipids, including large increases in the ether lipids. The exosomes released from these cells still had a similar ratio of PS 18:0/18:1 to the very-long-chain SM as in exosomes isolated from untreated cells^49^. Furthermore, a recent study of exosomes purified from urine of 15 prostate cancer patients and 13 healthy volunteers^50^ revealed a lipid composition different from that observed in PC-3 cells, but PS 18:0/18:1 in the inner leaflet could still theoretically occupy 78 ± 18% (mean ± SD) and 66 ± 22% of the area covered by the very-long-chain sphingolipids in the outer leaflet in patients and controls, respectively^50^. It should be noted that the cholesterol content has been reported to be above 40% of total lipids in several exosome preparations^45^. In the studies discussed above the cholesterol content was 44% of total lipids in exosomes released from PC-3 cells^11^ and 63% of total lipids in the urinary exosomes^50^. The last number is close to the maximum solubility reported for cholesterol in model membranes^51^.

As mentioned above, initial molecular dynamic simulation studies indicated a hand-shaking between the very-long-chain (C24) SM species and PS 18:0/18:1^11^. Later, a simulation study using 14 asymmetric lipid membranes^52^, demonstrated that in asymmetric models the very-long-chain N-amidated fatty acyl groups of SM were able to penetrate deeply into the inner leaflet. Interestingly, the conformational order of these amide-linked acyl chains was found to increase as they penetrated into the opposing leaflet, revealing that the acyl chains interact with lipids in the inner leaflet. Furthermore, cholesterol was shown to modulate the effect of SM interdigitation by influencing the conformational order of lipid chains in the inner leaflet^52^. The strongest interdigitation was observed between SM d18:0/24:0 and PS 18:0/18:1 (it should be noted that also PS 16:0/18:1 was included in that study). Notably, the interaction between these two species was the only one that increased in the presence of cholesterol. The strongest interdigitation was observed when there was a little more cholesterol in the SM containing leaflet than in the inner leaflet^52^.

**Fig. 3:**
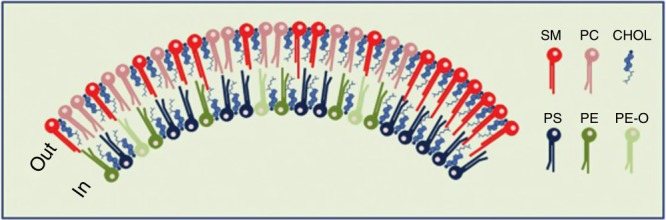
Schematic model of the lipid bilayer of exosomes. The number of lipid molecules (excluding cholesterol) shown in the outer (29) and inner (21) leaflet is close to the ratio for the outer and inner surface of exosomes with a diameter of 70 nm. The lipid composition of the membrane in this simplified illustration is based on the quantitative lipidomic data reported for 22 lipid classes of exosomes excreted from PC-3 cells^11^, i.e.,16 SM, 13 PC, 12 PS, 6 PE, 3 PE O (PE ethers) and 39 molecules of cholesterol (assuming a close to symmetric distribution of cholesterol between the two leaflets). In the right part of the membrane, a possible handshaking between the very-long-chain sphingolipids in the outer leaflet and PS 18:0/18:1 in the inner leaflet in the presence of cholesterol is illustrated. In the rest of the membrane, the lipids are distributed more or less evenly. Nine out of the 16 SM molecules shown contain a very-long-chain N-amidated fatty acyl group in accordance with the data published^11^. The figure is reproduced from ref. ^45^

The lipidomic analyses and the molecular dynamic simulation studies discussed above suggest a co-sorting and hand-shaking between the sphingolipids in the outer leaflet and PS 18:0/18:1 in the inner leaflet of exosomes. The fact that PS 18:0/18:1 is a major lipid species in exosomes excreted from many cell types led us to propose the model shown in Fig. 3, and investigate the membrane composition of other structures. It turns out that there are major similarities between the lipid composition of exosomes, HIV particles and DRMs^45^, and there is a high enrichment of PS, SM and cholesterol from the mother cell to both the HIV particles^53^ and the DRMs^54,55^. Moreover, PS 18:0/18:1 was the dominant PS in these preparations (PS 36:1 quantified in the HIV particles)^53^. Assuming that HIV particles have a diameter of 120 nm^53^, the PS 36:1 in the inner leaflet could theoretically cover ~60% of the very-long-chain sphingolipids in the outer leaflet, indicating that there could be a similar hand-shaking between the very-long-chain sphingolipids and PS 18:0/18:1 also in HIV particles^45^. It has for many years been stated that DRMs are highly enriched in saturated phospholipids. This may be due to the fact that DRMs used in an early and detailed study of their lipid composition were reported to be enriched in saturated phospholipids, the caveat that “a phospholipid was deemed saturated if containing no more than one double bond”^55^ might often be overlooked. It should also be noted that recycling endosomes have been shown to be enriched in SM, PS and cholesterol, but lipid species were not reported in that study^56^.

In summary, it appears that interaction between long-chain and especially the very-long-chain sphingolipids in one leaflet and PS 18:0/18:1 in the opposing leaflet is important for cellular membranes, not only for exosomes. Data showing interactions between PS 18:0/18:1 and cholesterol^57^ are discussed below. One may wonder why C18:1 (oleic acid; cis-9) in PS seems to be important for such interactions. Is this due to the fact that this double bound is located approximately in the middle of the inner leaflet, and close to the end of the very-long-chain N-amidated fatty acyl group penetrating from the other leaflet? Is the position of this double bound also important for cholesterol induced packing of cell membranes? It should be noted that cells contain much more PS 18:0/18:1 than e.g. PS 16:0/18:1 or PS 18:0/16:1 (Table 2).

**Table 2:** Percent of PS 18:0/18:1, PS 16:0/18:1 and PS species with two saturated FAs

Cell line	Number of PS species reported	PS 18:0/18:1 (% of total PS)^a^	PS 16:0/18:1 (% of total PS)^a^	Saturated PS (% of total PS)^b^	Refs.
PC-3	30	~33	~11	<2^c^	^11^
HEp-2	20	~60	~7	<5^d^	^10^
PSA3 (CHO)	9	~45^e^	~2^f^	<5^g^	^63^
HeLa	30	~22^e^	~14^f^	<3^h^	^53^
MT4	30	~29^e^	~3^f^	<1^h^	^53^
A549	10	~40^e^	~24^f^	<3^g^	^90^
Mouse fibroblasts	15	~51^e^	~11^f^	<2^i^	^91^

^a^The values given are estimated from the data reported (partly given only in figures) in the publications referred to

^b^The maximum amount of saturated PS species is estimated. Thus, if data for PS 32:0, PS 34:0 or PS 36:0 are not reported it is assumed that they are present in amounts close to the lowest value reported for PS species

^c^~1% of PS 16:0/18:0 and <0.05% of both PS 16:0/16:0 and PS 18:0/18:0

^d^~3% of PS 16:0/18:0 and very small amounts of PS 16:0/16:0 and PS 18:0/18:0 were detected

^e^Assuming all PS 36:1 is PS 18:0/18:1

^f^Assuming all PS 34:1 is PS 16:0/18:1; thus no PS 18:0/16:1

^g^PS species with two saturated FAs were not reported

^h^PS 36:0 and PS 40:0 were quantified; PS 32:0 or PS 34:0 were not reported

^i^PS 32:0 was the only species reported with two saturated FAs.

### Clustering of PS in one membrane leaflet

To our knowledge, a study published in 2011 was the first to indicate a possible interaction between sphingolipids in the outer leaflet and PS in the inner leaflet. The groups of Grinstein and Parton demonstrated topologically distinct cellular pools of PS^58^, using a PS-binding fluorescent probe and light microscopy, as well as electron microscopy (EM) after gold immune-labelling of this probe. They showed that PS on the cytosolic side of the plasma membrane forms clusters with a diameter of ~11 nm. PS clusters were also observed at the trans-Golgi network and at endocytic organelles. Importantly, PS clusters were observed at 60–80 nm vesicular profiles with the morphology of caveolae, which are known to be enriched in sphingolipids and cholesterol and can be regarded as raft-like structures^9,59^. Very recently another study showed that PS in fact dictates the assembly and dynamics of caveolae, indicating that membrane leaflet coupling is a prerequisite for their formation^60^. Grinstein and colleagues also showed that polarisation of PS into the bud of *Saccharomyces cerevisiae* was required for proper Cdc42 localisation and for development of cell polarity^61^. However, whether this polarisation is associated with membrane leaflet coupling has to our knowledge not been investigated.

### Clustering of PS in the inner leaflet and coupling to GPI

The Mayor group has shown that glycosylphosphatidylinositol (GPI)-anchored proteins (GPI-AP) could form nanoscale domains in living cell membranes^62^. More recently the same group (Raghupathy et al.) reported clustering and interaction between analogues of GPI-AP in the outer leaflet and PS in the inner leaflet of the Chinese hamster ovary (CHO) cell line PSA3, which carries a mutation in the PS synthase 1 gene^63^. Most of the reported experiments were performed using synthetic fluorescent GPI-AP analogues carrying glucosamine inositol linked phosphatidic acid (PA) with a fluorescent label linked to glucosamine, i.e. without any protein part. The clustering of the GPI analogue was reduced when cholesterol was extracted with methyl-β-cyclodextrin or by disrupting actin activity. Based on these data the authors concluded that both cholesterol and actin were important for this clustering^63^. In such experiments, it is however difficult to exclude that removal of cholesterol induces secondary effects that might be affecting the clustering. The clustering was observed when the lipid part of the GPI analogue contained two saturated long-chain fatty acyl chains (16:0/16:0 and 18:0/18:0 were used), but not when it contained two unsaturated (18:1/18:1) or short chains (8:0/8:0). Thus, the clustering was observed with GPI-AP analogues having fatty acyl chains close to those found in mature endogenous GPI-APs which contains an alkyl chain in the *sn-1* position^64^. Moreover, Raghupathy et al.^63^ showed that PS in the inner leaflet was required for this clustering, whereas PI(4,5)P_2_ was shown not to be involved.

By using a protein construct that connect PS to actin they could recruit endogenous GPI-APs in the outer leaflet, thus indicating that actin filaments at the inner leaflet have the capacity to link to PS and immobilise and stabilise local lipid-ordered domains in the outer leaflet of the plasma membrane^63^. There are some similarities between those observations and some earlier reports that cross-linking of GPI-AP resulted in a cholesterol-dependent intracellular signalling^33,65^.

As mentioned above, it has for many years been assumed that phospholipid species in rafts mainly contain saturated fatty acyl groups, and in most of the studies performed by Raghupathy et al.^63^ they compared the results obtained using PS with two saturated fatty acyl groups with those obtained with PS 18:1/18:1. Atomistic molecular dynamic simulation studies with asymmetric bilayers (GPI-analogues in one leaflet and PS species in the other leaflet) showed the strongest transbilayer interdigitation obtained with the GPI analogue carrying two fatty acyl groups with C16:0 and with PS 18:0/18:0; the interdigitation was much less when exchanging PS 18:0/18:0 with PS 18:1/18:1 or PS 12:0/12:0. Furthermore, Raghupathy et al.^63^ studied the effect of different PS species on the GPI-AP clustering; various PS species were added to the medium, and shown to be incorporated into the inner leaflet of the plasma membrane. Also when measuring the clustering of endogenous GPI-AP on the cell surface by adding PS species in the presence of a PLA2 inhibitor, PS 18:0/18:0 was much more effective than PS 18:1/18:1 or PS 12:0/12:0^63^. For these clustering experiments they also added PS 18:0/18:1 as they found PS 36:1 to be the dominant PS species in these cells. Since PS 18:0/18:1 gave a similar effect on clustering of GPI-AP as PS 18:0/18:0, it was concluded that long-chain PS species were needed to obtain this clustering^63^. As discussed further below, none of the 9 PS species reported to be present in PSA3 cells contained two saturated FAs (Table 2). Raghupathy et al.^63^ therefore suggested a general mechanism for the generation of cholesterol-dependent and actin-mediated lipid nanoclusters of GPI-APs and long-chain PS species in biological membranes. Their data for clustering of the GPI-AP analogues and PS in the plasma membrane fit well with the proposal of transbilayer coupling of sphingolipids and PS. Although interleaflet coupling was observed with C16 and C18 fatty acyl groups in the GPI-AP study^63^ and with SM with 16 carbon atoms in the N-amidated fatty acyl group in the exosome study^52^, much stronger interleaflet coupling was observed in the exosome study with the very-long-chain SM containing 24 carbon atoms.

### PS species found in cells

Considering the role of PS species in interleaflet coupling, it is of interest to quantify PS species in various cell lines. In Table 2 we summarise such data for seven cells lines, including all cell lines discussed in this review, in addition to three other cell types, i.e., MT4 (a human T-cell leukaemia cell line), A549 cells (a human alveolar basal epithelial cell line) and mouse fibroblasts. Furthermore, we have included data for PS 16:0/18:1 as the discussion in the next chapter shows that it is not only the C18:1 in the *sn-2* position of PS that is of importance, but also that the length of the saturated fatty acyl group in the *sn-1* position plays a role. Table 2 reveals that PS 18:0/18:1 is the dominating PS species in all these cell lines and that there is very little of PS species with two saturated fatty acyl groups. The quantification of other phospholipid classes in the publications listed in Table 2 and other studies reviewed in^66^, shows very small amounts of lipid species with two saturated fatty acyl groups. Quantitative analyses of lipid species of other cells are warranted to see if the species distribution shown in Table 2 is representative for most cell types. With the present knowledge about lipid species in cells, we conclude that the view that lipid rafts contain large amounts of phospholipids with two saturated fatty acyl groups should be questioned, including recent models containing only saturated PS species in the inner leaflet of rafts^17^.

### The importance of PS 18:0/18:1 and interactions with cholesterol

Maekawa and Fairn^57^ demonstrated that PS and especially PS18:0/18:1 is important for the proper transbilayer distribution of cholesterol. They used a fluorescent probe, D4H, which recognises cholesterol in the cytosolic leaflet of plasma membrane and organelles. This enabled them to estimate the distribution of cholesterol in the inner and outer leaflet of the plasma membrane of PSB-2 cells. These cells have only 11% of PS synthase activity compared to the CHO cells they were derived from, which resulted in 80% less PS in the PSB-2 cells than in the CHO cells. The transbilayer distribution of cholesterol in the plasma membrane was disturbed in the PSB-2 cells as relatively more cholesterol was present in the outer compared to the inner leaflet of the plasma membrane. The balance between cholesterol in the two leaflets could be normalised by supplementing the medium with PS, but not by adding PE or PC. Thus, their data are consistent with a redistribution of cholesterol from the inner to the outer leaflet of the plasma membrane when the PS level of the plasma membrane is reduced.

These authors also used giant unilamellar liposomes (GUVs) to study phase separation of cholesterol and PS^57^. Phase separation was achieved when using PS 18:0/18:1, but not when using PS 16:0/18:1, PS 18:1/18:1 or PS 16:0/18:2. Furthermore, PS 18:0/18:1 was the most efficient of these PS species in shielding cholesterol from being oxidised by cholesterol oxidase, and PS 18:0/18:1 also provided a better protection against cholesterol oxidation than egg SM, brain PI(4,5)P2, PC 18:0/18:1, PE 18:0/18:1 and PA 18:0/18:1. Thus, this data represent another example of the importance of PS 18:0/18:1, and indicates a role for interactions between PS 18:0/18:1 and cholesterol^57^. A summary of data obtained for PS 18:0/18:1 with those obtained for other PS species is given in Table 3.

**Table 3:** Comparison of data obtained for PS 18:0/18:1 with those obtained for other PS species

Methods used to generate data	Conclusions
MS analyses of cells	PS 18:0/18:1 is by far the most abundant PS species in most cell lines (see Table 1).
MS analyses of exosomes	PS 18:0/18:1 constitute ~40% of total PS species in exosomes released from PC-3 cells and could occupy ~80% of the area of the inner leaflet covered by the very-long-chain sphingolipids in the outer leaflet^11^.
Molecular dynamic simulation studies of the interdigitation between PS and sphingolipids	PS 18:0/18:1 was the only species giving increased interdigitation with SM d18:1/24:0 in the presence of cholesterol, and interdigitation was stronger with PS 18:0/18:1 than with PS 16:0/18:1^52^.
Shielding cholesterol from cholesterol oxidase	PS 18:0/18:1 better than PS 16:0/18:1, PS 18:1/18:1 and PS 16:0/18:2^57^.
Clustering with cholesterol in liposomes	PS 18:0/18:1 better than PS 16:0/18:1, PS 18:1/18:1 and PS 16:0/18:2^57^.
Clustering of GPI-AP	PS 18:0/18:1 similar to PS 18:0/18:0; better than PS 18:1/18:1 and PS 12:0/12:0^63^.

It was also recently reported that interactions between PS and cholesterol is important for control of membrane curvature^67^. It was proposed that cholesterol associates with PS to form clusters where the headgroups of PS are sufficiently separated to limit the curvature otherwise caused by the negative charges of PS^67^. Also, addition of PS 10:0/10:0 was sufficient to increase endocytosis, and removal of cholesterol with methyl-β-cyclodextrin resulted in PS-rich tubules emanating from the plasma membrane^67^.

### PS-binding proteins involvement in transport and signalling

As several lines of evidence support that PS can be clustered in the inner leaflet of the plasma membrane^57,58,61,63^, one may wonder if PS clustering is important to increase interactions between PS and several cytosolic PS-binding proteins. It should be kept in mind that translocation of proteins from the cytosol to the membrane may depend on the PS density in the binding area, such that it is not only the binding affinity between one PS molecule and the PS-binding protein that is important, but the high avidity created by the accumulated strength of interactions occurring close to each other in the membrane. Also the observation that HeLa cells have a significant fraction of PS with limited mobility, and that cortical actin contributes to the confinement of PS in the plasma membrane^68^ suggests that “immobilisation” of a fraction of PS could be due to binding of other cytosolic proteins as well. Although several PS-binding proteins are known^34,69^, more information is needed about this type of proteins and their interaction with PS in order to evaluate their importance for intracellular signalling. A search for proteins (SMART-EMBL database) with pleckstrin homology (PH) domains revealed 259 verified PH-containing proteins and 696 proteins containing the predicted sequences of PH domains thus being candidates for binding to PS. It is not our goal to discuss in detail what is known about PS-binding proteins, but Table 4 contains examples of PS-binding proteins which may contribute to intracellular transport or signalling and perhaps be influenced by PS clustering.

**Table 4:** Examples of PS-binding proteins

Proteins	Function
Rho GTPases	A family of 20 proteins in mammals controlling endocytosis, cell migration, cell progression and morphology^92,93^. Cdc42^61^ is discussed in the main text. It has been proposed that the size of the positive charge in the C-terminal area of Rho1 and Cdc42 is important for the specificity of binding to PS^94^. It is not known how many of the Rho GTPases that bind PS.
Rab GTPases and Rab11-FIPs^a^	Distinct associations between the PS-probe LactC2, Rab GTPases and Rab11-FIPs, suggesting that C2 domains are not the sole mechanism for Rab11-FIPs to associate with PS-containing membranes^95^.
K-Ras	K-Ras (see^48^ for a review) is a GTPase and an early player in many signal transduction pathways. The protein is prenylated and bound to membranes^96^. The KRAS-BRAF-MEK pathway requires the presence of both PS and the lysine-rich C-terminus of KRAS^97^. PS is important for binding and nanoclustering of K-Ras at the plasma membrane^98^. The highest colocalization between K-Ras and PS observed in BHK cells is with PS species containing C18:1 in the *sn-2* position^48^.
Protein kinase C	Enzyme family controlling the function of other proteins through phosphorylations^99^.
Akt (Protein kinase B)	Activation of Akt mediates downstream responses through protein phosphorylations; Akt activation relies on binding to both PI(3,4,5)P3 and PS^100^.
EHD1	Transport through recycling endosomes^81^ and further to the Golgi apparatus^101^.
Evectin-2	Retrograde transport through endosomes to the Golgi apparatus^80^.
ATP9A	Flippase colocalizing with PS in endosomes^82^.
Synaptotagmin	Ca^2+^-triggered PS binding has multiple functions in exocytosis, including stabilisation of open fusion pores by retarding the rate of fusion pore dilation^102^.
Cavins	Cavins contain PS-binding domains; cavins are able to associate into oligomers and are present in caveolae^103^ which are enriched in PS in the inner leaflet^58^. Cavin-1 forms polyhedral lattices on PS-containing liposomes^104^. PS is required for cavin-1 and caveolin-1 to form stable caveolae in vivo^60^.
Caveolins	Caveolins are together with cavins important for formation of caveolae and can as cavins form oligomers^103^. Since caveolins also bind to cholesterol^105^, one may speculate if caveolins together with cavins induce clusters of PS. Knock-down of caveolin-1 resulted in reorganisation of PS within the plasma membrane^106^.
Myosin-1A	Myosin-1A binds to liposomes composed of PS and PI (4,5)P2 and colocalized with PS-binding and PI(4,5)P2-binding probes. Only the PS-binding probe (Lact-C2) reduced brush border targeting of a C-terminal domain of myosin-1A^107^. Myosin-1A is a candidate for linking actin and PS.
Spectrin	PS-binding sites identified^108^; spectrin plays an important role in maintenance of plasma membrane integrity and cytoskeletal structure^109^. Spectrin is a candidate for linking actin and PS.
Proteins transporting PS from ER to the plasma membrane	Oxysterol-binding proteins in *Saccharomyces cerevisae* bind PS and contribute to PS transport to the plasma membrane^110^. In HeLa cells the human homologues ORP5 and ORP8 mediate ER-plasma membrane contact and deliver PS from the ER to the plasma membrane^111^.

^a^FIPs: family interacting proteins.

### PS in intracellular membranes

Our discussion so far has to a large extent focused on the structure of the plasma membrane, but several of the issues discussed are also relevant for other cell membranes. PS was found in the luminal side of ER, the Golgi complex and mitochondria^58^, and PS is also exposed to the cytosol during transport from the trans-Golgi to the plasma membrane, as well as in endosomes and lysosomes^70^. The flipping of PS to the cytosolic leaflet of trans-Golgi has been shown to be due to Arl1 activation of the flippase Drs2 in the trans-Golgi network, and has recently been reviewed^71^. It should be noted that sphingolipids and cholesterol are selectively enriched in membranes from trans-Golgi to the plasma membrane^72,73^, and it has been proposed that rafts containing cholesterol and sphingolipids are important for sorting of proteins from the trans-Golgi to the plasma membrane^74^. Thus, flipping of PS to the cytosolic side of the trans-Golgi^71^ and interleaflet coupling may be important for sorting of at least of some of these proteins.

For more information about the asymmetry of PS, the importance of flippases, floppases and scramblases as well as other probes for intracellular detection of PS, we refer to the following articles: asymmetry^1,13^, flippases etc.^75,76^, and PS-probes^75,77^. The formation and some functional aspects of PS have recently been reviewed^78^.

Discussions in the literature about interactions between protein and lipids in the plasma membrane and endosomal vesicles have until now mainly been related to interactions with PIPs. The rapid metabolism of PIPs and their importance for the function of Rab proteins make them suitable for regulating intracellular transport^79^. The headgroup of PS is not metabolised as fast as PIPs, but on the other hand PS is often present in much higher amounts than the PIPs.

There are examples of PS-binding proteins being important for intracellular transport, e.g., that evectin-2 is important for retrograde transport of cholera toxin from endosomes to the Golgi apparatus^80^, EHD1 is important for transport through recycling endosomes to the Golgi apparatus, and it has been suggested that EHD1 not only binds to PA but also to PS^81^. The flippase ATP9A, which is colocalized with PS in early and recycling endosomes, plays a crucial role for recycling from endosomes to the plasma membrane^82^. Thus, it is clear that PS can play a role for intracellular transport.

### Molecular dynamic simulation studies and membrane models

Recently molecular dynamic simulation has proven to be a useful tool to study membrane structures. Although we focused in this review on interleaflet interactions between the very-long-chain sphingolipids and PS 18:0/18:1, it is important to be aware of that interleaflet coupling is found in almost all membrane models. Thus, although we reported the highest interdigitation in asymmetric models with SM d18:1/24:0 and PS 18:0/18:1 in the presence of cholesterol, it should be noticed that interdigitation was measured both when using the shorter SM species with N-amidated C16:0, and with other lipids species and headgroups than PS 18:0/18:1^52^. Although a significant interdigitation has been observed in the symmetric models, a considerably stronger interdigitation was observed when using asymmetric models^52^, thus stressing the importance of using asymmetric membrane models, i.e. models that are more similar to biological membranes^1,13^. For more information about simulation studies we refer to recent reviews discussing cholesterol and sphingolipids in raft-like membranes^83^, the role of charged lipids in membrane structures^84^, and to two reviews discussing different theories of leaflet coupling and various methods used to study such interactions^47,85^. Of special interest for the present discussion is that these reviews all stress the importance of using asymmetric models and the ability of the very-long-chain sphingolipids to cross the membrane midplane.

In the two last-mentioned reviews^47,85^ there are also discussions about the possible contribution of interleaflet coupling due to membrane curvature, line tension, electrostatic interactions and cholesterol flip-flop between membrane leaflets. It has been suggested that the so-called cholesterol flip-flop may give important contributions to the leaflet coupling, but it is argued in the review by Nickels et al.^85^ that the flip-flop contributes more than a magnitude less than that due to the acyl chain interactions.

Also several interesting studies have been performed using vesicles with an asymmetric membrane. Thus, Chiantia and London^86^ measured diffusion (using fluorescent correlation spectroscopy) in each membrane leaflet of vesicles containing various SM species in the outer leaflet and various PC species in the inner leaflet. They observed the largest reduction of diffusion in the inner leaflet when using SM C24:0 in the outer one, indicating that this very-long-chain species gave the strongest coupling between the leaflets. The largest reduction of diffusion in the outer leaflet was obtained when the inner leaflet contained PC with one saturated fatty acyl group (either C14, C16 or C18) and one unsaturated fatty acyl group (C18:1)^86^. They also postulated that van der Waals interactions between the terminal portions of acyl chains from the two leaflets meeting near the midplane is likely to contribute to chain interdigitation effects. Such effects may thus be important for bilayer coupling between membrane areas containing mainly C16 or C18 fatty acyl groups. Later, the same group, using vesicles containing 37% cholesterol and asymmetric membranes with an SM-rich outer leaflet demonstrated the formation of ordered domains in the inner leaflet containing only PC with two unsaturated fatty acyl groups (PC 18:1/18:1)^87^. Thus, such interactions were demonstrated even with two unsaturated fatty acyl groups in the glycerophospholipids in the inner leaflet, i.e., at conditions expected to be less optimal for such interactions.

The knowledge of making asymmetric liposomes is relatively new and one should expect more studies using such liposomes in the future. It would be useful if one could develop an asymmetric vesicle system with cholesterol, with defined species of both a glycosphingolipid such as Gb3 in the outer leaflet and PS in the inner leaflet and include a reporter kinase to determine whether crosslinking of Gb3 could induce a transmembrane signalling similarly to what was reported by Iwabuchi et al.^88^.

Despite that asymmetric membrane models are more similar to biological membranes, most studies are still performed with symmetric models containing only one or a few lipid species. Moreover, the lipid species used in such models are often present in very low amounts in cells. These models typically contain identical saturated, monounsaturated or diunsaturated fatty acyl groups, whereas mammalian cells contain high levels of phospholipid species with one saturated and one unsaturated fatty acyl group^10,11,66^. Even fatty acyl groups much shorter than those found in biological membranes are often used in such models. Thus, a critical discussion of the biological significance of the data obtained in such studies should be included, but are most often lacking. Although the use of giant plasma membrane vesicles have several advantages compared to liposomes regarding similarity with cellular membranes^66^, it should be noted that these large membrane vesicles have been reported to lose their PS asymmetry^89^, a change that might affect membrane properties, including interdigitation.

## Conclusion

Here we have summarised recent studies which demonstrate coupling between the two leaflets in cellular membranes with a special focus on the importance of the ability of PS 18:0/18:1 present in the inner leaflet of the plasma membrane to interact with very-long-chain sphingolipids in the outer leaflet in a cholesterol dependant manner. The role of coupling between the two membrane leaflets for association with cytosolic proteins and intracellular signalling is discussed. Membrane domains referred to as lipid rafts are often described to be enriched in phospholipid species with two saturated fatty acyl groups. However, such saturated species normally constitute a very small fraction of cellular lipids, and the view that lipid rafts mainly contain phospholipids with two saturated fatty acyl groups should therefore be reconsidered. PS 18:0/18:1 is the major PS species in many cell types and the only species where addition of cholesterol in molecular dynamic simulation studies revealed increased interleaflet coupling with sphingolipids. Although more has to be learnt, the molecular properties and abundance of PS 18:0/18:1 suggest a unique role for this lipid species in cell membranes. Some ideas for future studies in this field are included in Box 1.

Box 1 Recommendations for future studies

- Quantification of hundreds of lipid species can now be performed in one sample using MS analyses. It is important that such quantitative data are made available for everybody, as it may take several years before we are able to interpret these data.
- The methods used for sample preparation and for lipid analyses need to be better documented and validated such that scientists can trust that the data are reproducible.
- People should carefully evaluate if their lipid data indicate the presence of impurities in their samples. For example, large amounts of triacylglycerols or cholesteryl esters in preparations of cellular membranes indicate the presence of lipid droplets.
- When studying the effect of adding lipids to cells it is important that not only the amount and lipid class is given, but the composition of lipid species.
- New studies should clarify if plasma membranes and cells in general contain as much PS 18:0/18:1 and as little phospholipids with two saturated fatty acyl groups as discussed in this review.
- Future studies should address whether the importance of PS 18:0/18:1 is due to that the double bond of C18:1 is in the middle of the membrane leaflet, and if that provides optimal interaction with cholesterol.
- New studies should aim at clarifying if interdigitation between sphingolipids and lipids in the other leaflet is stronger with PS than with other lipids. If yes, why?
- One would like to know if the large amount of sphingolipids with N-amidated C24:1 is due to that the double bond is just in the middle of the lipid bilayer and why that is important.
- To make synthetic lipid layers as similar to biological membranes as possible—including for molecular dynamic simulation studies—it is important to use asymmetric membranes and lipid species known to be present in high amounts in the membrane to be mimicked.
